# The prevalence of high-risk human papillomavirus genotypes and related risk factors among Iranian women

**DOI:** 10.25122/jml-2022-0031

**Published:** 2022-11

**Authors:** Maryam Hashemnejad, Roghieh Mirmajidi, Mitra Rahimzadeh, Mina Ataei

**Affiliations:** 1 Obstetrics and Gynecology Department, School of Medical Sciences, Alborz University of Medical Sciences, Karaj, Iran; 2 Social Determinants of Health Research Center, School of Public Health, Alborz University of Medical Sciences, Karaj, Iran; 3 Reproductive Biotechnology Research Center, Avicenna Research Institute, ACECR, Tehran, Iran

**Keywords:** papillomaviridae, prevalence, risk factors, uterine cervical neoplasms

## Abstract

Human papillomavirus (HPV) infection, which often includes high-risk genotype infection, is one of the leading causes of cervical cancer. This cross-sectional research included 503 Iranian women referred to the gynecology clinic of Kamali Hospital in Karaj, Iran, for routine cervical cancer screening between 2020 and 2021. Cervical specimens were collected from all participants with a special brush and transported to the laboratory for cervical cytology diagnosis. Overall HPV incidence among Iranian women was 39.96%, of which 23.06% had high-risk HPV genotypes and 9.7% had low-risk HPV types. The risk associated with HR-HPV types was considerably associated with employment and marital status.

## INTRODUCTION

Cervical cancer is the fourth cancer most often identified among females [1]. In 2018, there were 570,000 complaints of cervical cancer, and 310,000 deaths were reported [2]. During the 20^th^ century, cervical cancer was the most reported and known cause of mortality in females [2]. Although cervical cancer has been moderately well controlled in many high-income countries, it is still regarded as the known cause of cancer-related mortality in women in many low-income and lower-middle-income nations [2].

Papillomaviruses are double-stranded deoxyribonucleic acid (DNA) viruses detected in animals and humans. Over 200 human papillomavirus genotypes (HPV) have been recognized so far, and nearly 40 genotypes infect genitalia [3]. Genital HPV infection is related to the growth of cervical neoplasia, cervical cancer, anogenital warts, and other anogenital cancers [4]. Based on HPV associations with malignant or benign proliferative lesions, categorization might be either high-risk HPV (HR-HPV) group or oncogenic, or low-risk HPV (LR-HPV) group or non-oncogenic [5]. HR-HPV was recognized as a fundamental reason for the growth of pre-invasive and invasive cancers of the lower genital tract, where cancer of the cervix is dominant [6]. Although genital warts caused by LR-HPV do not participate in increased morbidity and mortality, it leads to anxiety, uneasiness, pain, and shame due to medical costs and psychological distress [7]. Numerous HPV infections are often asymptomatic and clear in the host immune cell within one to two years after infection [8].

Unprotected sexual intimacy is the major risk factor related to the attainment and durability of HPV infection and the growth of HPV-associated cancers [9]. These unprotected sexual activities include the age of first sexual activity, number of sexual partners, and the total figure of lifetime sexual partners [9]. Other risk factors are oral contraceptive use, gravida status, socioeconomic status, cigarette smoking, and co-infection with a human with sexually transmitted diseases (STDs) and immunodeficiency virus (HIV) [7, 9]. The difference in HPV incidence in different areas of the globe suggest the possibility of other HPV-infection risk factors [10]. A greater number of sexual partners were seen in individuals infected with high-risk HPV and multi-type HPV compared to low-risk type and single-type, respectively [10].

It is important to study the predictors of HPV infection in high-risk areas to address the gaps in healthcare, giving special consideration to the differences in sexual behavior among men and women and people from various racial origins. Previous studies have focused on the incidence and genotype distribution of HPV infection among Iranian females [11, 12]. Although a strong relationship exists between HPV risk factors, HPV infection, and the risk of cervical cancer, to date there is no complete study that evaluates the combination of the aforementioned factors in Iranian inhabitants. Therefore, the present study was performed to evaluate the prevalence of HR-HPV genotypes and identify related risk factors among Iranian women referred to the gynecology clinic of Kamali hospital, Karaj, Iran.

## MATERIAL AND METHODS

### Study population

This cross-sectional research included 503 Iranian women referred to the Gynecology Clinic of Kamali Hospital in Karaj, Iran, for routine cervical cancer screening between 2020 and 2021. Sexually active, 15–70 years old, from the Iranian population, having clinical symptoms including genital warts, intermenstrual bleeding, vaginal discharge, pain during intercourse and bleeding after intercourse, and or having come for annual visits were considered as inclusion criteria. Exclusion criteria included abnormal vaginal bleeding, obvious mass in the cervix, hysterectomy or management of potential cervix cancer, obstetrical/gynecological emergency, pregnancy, and mental impairment. Women who agreed to participate were invited to a brief meeting and a gynecological examination, comprising collecting samples for cytology diagnoses and HPV tests.

### Data collection

Interviews were done in a private room to obtain the medical history, demographic and behavioral characteristics of participants. Questions included demographic profile and HPV-related risk factors, including employment status, level of education, breastfeeding status, marital status, menstrual cycle status, gravida status, post-coital bleeding, number of sexual partners, hookah use, cigarette status, and reason for referral [7, 9, 13]. The interviewer reviewed the questionnaire with the participant to confirm that all questions were answered correctly.

### Clinical Examination

Participants completed the routine gynecological examination after the administration of the questionnaire. A trained gynecologist collected cervical samples using cytobrush at the intersection of the endocervix and the ectocervix. Two midwives participated in observing the collection of samples. The samples were kept in a protective solution abiding by the manufacturer's guidelines and transported to the laboratory for Papanicolaou (Pap) smear or cervical cytology diagnosis. Cervical cytology smears were examined by a cytopathologist who did not know the participant's HPV DNA test results. Cytological results were defined as high-grade intraepithelial lesions (HSILs), low-grade squamous intraepithelial lesions (LSILs), atypical glandular cells (AGCs), atypical squamous cells of undetermined significance (ASCUS), cervical intraepithelial neoplasia (CIN), koilocytic change, and chronic cervicitis [14, 15].

### HPV Analyses

Samples for HPV analysis were collected using cervical swabs, placed in a sterile tube, and transported to the test center for HPV DNA testing using a PCR-based method. The presence of 36 HPV genotypes including 18 LR–HPV genotypes (HPV 6, 11, 40, 42, 43, 44, 54, 55, 61, 62, 67, 69, 70, 71, 72, 81, 84, and 89), 18 HR-HPV (HPV 16, 18, 26, 31, 33, 35, 39, 45, 51, 52, 53, 56, 58, 59, 66, 68, 73, and 82) were evaluated [12, 16].

After obtaining the HPV DNA test and cytology results, women with positive HR-HPV DNA or a Pap result of ASCUS or higher were sent to a gynecologist for further assessment or colposcopy examination.

### Statistical analysis

Statistical analysis was done using SPSS 21.0 statistical software package (SPSS Inc, Chicago, IL, USA) and GraphPad Prism v.7.0 (GraphPad, San Diego, USA). P-values<0.05 were considered statistically significant.

## RESULTS

607 participants completed the inclusion criteria and were included in this research, out of which 503 participants completed the study, and their data were analyzed. Table 1 describes the characteristics of the participants, including gravid status, breastfeeding, menstrual cycle, type of delivery, polyamory, post-coital bleeding, substance abuse, high-risk sexual behavior, hookah use, cigarette smoking, sexual onset, and age at first pregnancy.

**Table 1:** Demographic and obstetrics characteristics of the participants.

Characteristics		F (%)	Characteristics		F (%)
Gravid	0	54 (10.7)	Polyamory	Yes	22 (4.4)
				No	481 (95.6)
	1	87 (17.3)	Post-coital bleeding	Yes	92 (18.3)
	2	140 (27.8		No	409 (81.3)
	3–5	187 (37.2)	Cigarette	Yes	42 (8.3)
	≥6	35 (7)		No	458 (91.1)
Breastfeeding	Yes	224 (44.5)	Substance abuse	Yes	2 (04)
				No	496 (98.6)
	No	269 (53.5)	High-risk sexual behavior	Yes	24 (4.8)
				No	477 (94.8)
Menstrual cycle	Normal	426 (84.7)	Hookah	Yes	27 (5.4)
	Menorrhagia	30 (6)		No	475 (94.4)
	Oligomenorrhea	47 (9.3)	Age at first sex (years) mean±SD		20.2±5.2
Type of delivery	Cesarean	95 (18.9)			
	Vaginal delivery	314 (62.4)	Age at first pregnancy (years) mean±SD		22.1±5.2
	Both	31 (6.2)			

Table 2 represents the frequency distribution of the participants' reasons for cytology examination and HPV test. The most common reason for cytology examination and HPV test was annual screening (67.5%). Other reasons were abnormal vessels (6.2%), chronic cervicitis (9.7%), post-coital bleeding (11.9%), and wart (4.6%).

**Table 2:** Frequency distribution of reasons for cytology and HPV test in the participants.

Variable	Frequency	Percent
Annual Screening	340	67.5
Abnormal vessel	31	6.2
Chronic cervicitis	49	9.7
Post-coital bleeding	60	11.9
Wart	23	4.6
Total	503	100.0

### Prevalence of HPV

The results of PCR demonstrated that the overall HPV prevalence among Iranian women was 39.96% (201/503), from which 23.06% (116/503) had HR-HPV genotypes, 9.7% (49/503) had LR-HPV genotypes and 7.2% (36/503) had both HR-HPV and LR-HPV genotypes.

### Cytology results based on HPV infection

Table 3 shows the distribution of cytology results based on HPV infection. ASCUS was the most common abnormal finding from cytology results. 54.47% of the participants had ASCUS, 0.19% koilocytic change, 7.5% CIN1, 0.19% CIN2, 1.9% CIN3, 9.9% chronic cervicitis, 3.5% AGUS, whereas 22.06 women had normal cytology. Of the 392 cytology-positive women (77.93%), 67 individuals (17.09%) were HR-HPV positive. It was determined that 48 individuals (41.3%) with HR-HPV positive had normal cytology.

**Table 3:** Distribution of cytology results based on HPV infection.

Variable		HR-HPV		Total
		No F (%)	Yes F (%)	
Cytology results	Koilocytic change	1 (0. 25)	0	1 (0.19)
	CIN1	26 (0.06)	12 (10.3)	38 (7.5)
	CIN2	1 (0.25)	0	1 (0.19)
	CIN3	10 (2.5)	0	10 (1.9)
	ASCUS	231 (59)	41 (35.3)	274 (54.47)
	Chronic cervicitis	41 (10.5)	9 (7.7)	50 (9.9)
	AGUS	13 (3.3)	5 (4.3)	18 (3.5)
	Normal	63 (16.2)	48 (41.3)	111 (22.06)
Total		387	116	503

### Risk factors for HR-HPV types

Table 4 shows risk factors for HR-HPV positive compared to HPV negative in the study population. The Chi-square test revealed that the risk of having HR-HPV types was considerably related to employment status (p-value=0.003), level of education (p-value=0.001), gravida status (p-value=0.003), breastfeeding status (p-value=0.002), marital status (p-value=0.009), number of sexual partners (p-value=0.04), hookah use (p-value=0.001) cytology result (p-value=0.001), colposcopy test result (p-value=0.005) and reason for referral (p-value=0.002). On the other hand, the results demonstrated that the risk of having HR-HPV types was not significantly related to the status of the menstrual cycle (p-value=0.87), post-coital bleeding (p-value=0.27) and smoking cigarettes (p-value=0.58).

**Table 4:** The association of risk factors with high-risk HPV.

Risk factors		Negative HPV F (%)	Positive HPV F (%)	P-value
Employment	Housewife	35 (7)	94 (18.7)	0.003**
	Worker	351 (69.9)	22 (4.4)	
Education	Primary or High school	206 (41)	44 (8.7)	0.001***
	Diploma	121 (24.1)	38(7.6)	
	University	60(12.01)	34(6.8	
Gravida	0	36 (7.2)	18 (3.6)	0.003**
	1–3	246 (48.9)	73 (14.6)	
	4–5	75 (14.9)	20 (4)	
	>5	30 (6)	5 (1)	
Breastfeeding	Yes	186 (37.8)	38 (7.7)	0.002**
	No	190 (38.6)	78 (15.9	
Menstrual cycle	Normal	326 (64.8)	100 (19.9)	0.87
	Menorrhagia	24 (4.8)	6 (1.2)	
	Oligomenorrhea	37 (7.4)	10 (2)	
Polyamory	Yes	13 (2.5)	9 (1.7)	0.04*
	No	387 (75)	107 (20.7)	
Marital status	Married	379 (75.3)	108 (21.5)	0.009**
	Single or Divorce	8 (1.6)	8 (1.6)	
Post-coital bleeding	Yes	67 (13.4)	25 (5)	0.27
	No	319 (63.8)	89 (17.8)	
Cigarette	Yes	31 (6.2)	11 (2.2)	0.58
	No	355 (71)	103 (20.6)	
Hookah	YES	10 (2)	17 (3.4)	0.001***
	No	376 (74.9)	99 (19.7)	
Pap smear test result	Koilocyte	271 (54)	-	0.001***
	CINI	77 (15.3)	-	
	CINII	38 (7.6)	-	
Colposcopy test result	Negative	288 (57.4)	71 (14.1)	0.005*
	Positive	98 (19.5)	45 (9)	
Reason for referral	Screening	264 (52.9)	72 (14.4)	0.002*
	Abnormal Vessel	29 (5.8)	2 (0.4)	
	Chronic Cervicitis	36 (7.2)	13 (2.6)	
	Post-coital bleeding	46 (9.2)	14 (2.8)	
	Wart	11 (2.2)	12 (2.4)	

* – P-value<0.05; ** – P<0.01; *** – P<0.001.

A logistic regression analysis was done to identify the risk factors for HPV. Hookah use (7.986 times), cytology result (CN1) (3.802 times), employment status (3.331 times), cytology result (CN2) (2.309 times), breastfeeding (2.506 times), and early age at first pregnancy (1.068 times) increased the chance of being HR-HPV positive (Table 5).

**Table 5:** The predictive role of several variables in HPV.

Risk factors			P-value	Exp(B)
Step 6^f^	Age at First Pregnancy		0.005**	1.068
	Employment Status		0.003**	3.331
	Breastfeed		0.001**	2.506
	Hookah		0.000****	7.986
	Cytology results	Koilocyte	0.652	0.820
		CIN1	0.003**	3.802
		CIN2	0.004**	2.309
	Constant		0.000	0.017

* – P-value<0.05; ** – P<0.01; *** – P<0.001.

## DISCUSSION

In this study, the overall incidence of HPV among Iranian women was 39.96%, of which 23.06% had HR-HPV genotypes, 9.7% had LR-HPV genotypes, and 7.2% had both HR-HPV and LR-HPV genotypes. In addition, our results revealed that the risk of HR-HPV genotypes was considerably associated with employment status, level of education, gravida status, breastfeeding status, marital status, number of sexual partners, hookah use, cytology test result, colposcopy test result, and reason for referral.

Cervical cancer is the 10^th^ leading cause of cancer mortality in women 15 to 44 years of age in Iran [17]. It is believed that persistent HR-HPV infection causes over 90% of cervix cancer [18]. HPV prevalence has been reported at 8.0% in Asia, 22.1% in Africa, 8.1% in Europe, and 11.3% to 20.4% in America [19]. Herein, the overall HPV occurrence was determined at 39.96%. In line with the present study, in 2021, Bitarafan et al. showed that the overall prevalence of HPV infection among 12076 Iranian women was 38.68% [12]. However, in the Bitarafan et al. study, the prevalence of HR-HPV infections was approximately 15%. The present study revealed a higher HR-HPV prevalence (23.1%) amongst Iranian females. In addition, in comparison with studies done in Asia, the rate of HR-HPV was higher than presented in Nepal (6.1%), India (10.3%), Indonesia (7.9%), Bangladesh (4.2%), Japan (17%) and China (21.07%) [16, 20]. It seems that the high positivity of HR-HPV in the present study was due to various inclusion criteria and the population profile with a higher rate of incidences of atypical cervical cytology. However, alterations in interaction and inadequate knowledge regarding sexual matters may be the main reasons for a high rate of HR-HPV prevalence infection in recent years.

One of the effective methods for cervix cancer screening for many years has been Pap smear or cervical cytology. The benefits of cytology are its low cost and simplicity [21]. Based on the results from the cytology report, ASCUS was the most common abnormal finding in a Pap test. 272 participants (54%) had ASCUS, from which 41 participants (15%) had HR-HPV genotypes. Our findings revealed that of the 390 cytology-positive women, 67 individuals (17.17%) were HR-HPV positive. In addition, it was determined that CN1 increased the chance of positive HR-HPV genotypes by 3.8 times and CN2 by 2.309 times. This result suggests a relationship between HR-HPV genotypes and cervical cancer risk. Several studies have shown that the incidence of HR-HPV genotypes is related to the severity of abnormal lesions in cytology [22, 23]. Recently, Salavatiha et al., in a meta-analysis of HPV prevalence among Iranian women, revealed that the overall HPV incidence was 9% in females with a healthy cervix and 55% in ASCUS [24]. Therefore, it is valuable to consider the diagnostic methods, percussion, and identification of individuals with this disease to prevent cervical cancer.

It is of great importance to recognize distinctive risk factors of HR-HPV genotypes, along with ways to minimize the associated risk factors and take necessary actions against them. In the absence of a complete study that combines all the risk factors in an Iranian population, we studied the association between employment status, level of education, gravida status, breastfeeding status, marital status, menstrual cycle status, post-coital bleeding, and many sexual partners, hookah use, cigarette status, Pap smear test result, colposcopy test result and reason for referral. The results revealed that the overall risk for HPV infection among Iranian women was associated with employment status, level of education, gravida status, breastfeeding status, marital status, number of sexual partners, hookah use, Pap smear test result, colposcopy test result, and reason for referral. Among these risk factors, hookah use (7.986 times), Pap smear test result (CN1) (3.802 times), employment status (3.331 times), Pap smear test result (CN2) (2.309 times), breastfeeding (2.506 times), and early age at first pregnancy (1.068 times) increased the chance of being HR-HPV positive.

Based on our results, hookah was the most common risk factor for HR-HPV positive. Traditional use of hookah is common in Iran. Even though society is well aware of the disadvantages and dangers of hookah, bearing in mind the tradition of smoking hookah in Iran, many see it as healthy and harmless. Finding nicotine and carcinogenic properties of tobacco (used in a hookah) in cervical mucus has led to the hypothesis that the mixture of tobacco and HPV occurs in the development and resistance of cervical lesions [25]. It has been shown that the chemical compounds of tobacco act as a carcinogen and cause a mitogenic effect on the cell, reducing immunokinases in the cervical epithelium [26]. Ferson et al. reported that the actions of natural killer cells of smokers were considerably reduced than those of non-smoking individuals [27]. In addition, the levels of immunoglobulin (IgA and IgG) were lower in those who smoke than in those who do not [27]. Therefore, immune suppression in smokers may play a crucial role in the greater incidence of HPV and HPV-related tumors [28]. Although the risk associated with HR-HPV types was not considerably associated with smoking cigarettes in this research, Eldridge et al. reported that cigarettes are a risk factor in the natural history of HPV [26]. The biological role that hookah and cigarettes play in HPV infection remains unknown, and limited association data exist on the relationship between the smoking of hookah and HPV infection. Based on our knowledge, this result demonstrated for the first time that the use of hookah is related to an increased risk of oncogenic HR-HPV.

The socioeconomic status of a person affects the occurrence of health-related activities. Individuals with low socioeconomic status may lack the ability to afford essential health care or may live in regions with limited access to health services, which can generate susceptibility to the growth of various diseases. We used employment status and level of education as indicators of the socioeconomic status of the women. Bad health-related quality of life in individuals who suffer from HPV-positive can be mostly related to employment status. Herein, the obtained results showed that employment status is related to the rate of HPV. As shown, the rate of HPV in working women was greater than in homemaker women. One explanation for this result may be wearing clothes for outdoor work and less hygiene during office hours. However, Kulhan et al. in Turkey showed no relationship between employment status and HPV infection in women [13].

Early sexual debut is related to a higher risk of HR-HPV infection [29]. Age at first marriage is frequently used to determine age at first sexual intercourse, and patients who have early sexual intercourse might get pregnant at an early age [29]. In line with the present study, it was shown that early age at first pregnancy surges the chances of high-risk lesions or cervical carcinoma and even raises the risk of HR-HPV infection [30]. During pregnancy, hormonal alterations and immune system modifications can be responsible for the variation of the normal nature of HPV infection. Debated findings on the risk of HPV infection in pregnant women exist [31]. Although a greater HPV occurrence has been presented in literature regarding pregnant women, some state that no dissimilarity exists among age-matched non-pregnant women [31]. More studies are needed to evaluate the association between age at first pregnancy and HPV infection.

Finally, this research revealed that the incidence of HPV was greater in women who were not breastfeeding. It was suggested that the incidence of HPV was greater in people with breast cancer [32]. Therefore, according to the present results, it can be concluded that due to the effect of breastfeeding on reducing breast cancer, it can also reduce HPV infection [33]. However, the influence of breastfeeding on HPV disease and, consequently, growth of cervical neoplasia and its exact mechanism remain unknown.

This study has several strengths. Firstly, according to our understanding, it remains the only research to report the relationship between socioeconomic status, especially employment status, hookah use, and HR-HPV infection in Iranian women. Secondly, this study included several females from the overall population. Given its size, we could analyze the relationship between numerous risk factors and the prevalence of HR-HPV infection with a relatively strong statistical power. However, many limitations exist that require attention. Firstly, this research was performed only on a case group of individuals who attended the gynecology clinic of Kamali Hospital in Karaj, Iran. Due to this, we are confident that our findings should not be generalized to all Iranian women. Secondly, a lack of data regarding males' sexual behavior is also an important risk factor for HPV infection and cervical infections in females.

## CONCLUSION

In conclusion, we observed that HR-HPV is highly prevalent among Iranian women. The main risk factors for HR-HPV disease are employment status, level of education, parity, breastfeeding status, civil status, figures of sex partners, hookah use, and Pap smear test result. Since HPV is a great risk factor for cancer of the cervix, considering the diagnostic methods, prevention and identification of individuals with this infection is a useful way to prevent this cancer. Hence, determining the HPV prevalence and related risk factors in major inhabitants of the Iranian community can develop and advance health policies applied by the government and health agencies.

## Personal thanks

We acknowledge all staff who worked at the gynecology clinic of Kamali Hospital in Karaj, Iran, for their great help in performing this study.
